# Effect of Early and Intensive Telephone or Electronic Nutrition Counselling Delivered to People with Upper Gastrointestinal Cancer on Quality of Life: A Three-Arm Randomised Controlled Trial

**DOI:** 10.3390/nu14153234

**Published:** 2022-08-07

**Authors:** Catherine E. Huggins, Lauren Hanna, Kate Furness, Mary Anne Silvers, June Savva, Helena Frawley, Daniel Croagh, Paul Cashin, Liang Low, Judy Bauer, Helen Truby, Terry P. Haines

**Affiliations:** 1 Department of Nutrition, Dietetics and Food, Monash University, Level 1, 264 Ferntree Gully Road, Notting Hill, VIC 3168, Australia; 2 Institute for Health Transformation, Deakin University, 221 Burwood Highway, Burwood, VIC 3125, Australia; 3 Department of Nutrition and Dietetics, Monash Health, 246 Clayton Road, Clayton, VIC 3168, Australia; 4 Department of Physiotherapy, Monash University, Level 3 Building G, McMahons Road, Frankston, VIC 3199, Australia; 5 Upper Gastrointestinal and Hepatobiliary Surgery Unit, Monash Health, 246 Clayton Road, Clayton, VIC 3168, Australia; 6 Department of Surgery, School of Clinical Sciences, Faculty of Medicine, Nursing and Health Sciences, Monash University, Level 5 Block E, 246 Clayton Road, Clayton, VIC 3168, Australia; 7 School of Human Movement and Nutrition Sciences, The University of Queensland, Level 2 Connell Building, St. Lucia, QLD 4072, Australia; 8 National Centre for Healthy Ageing, School of Primary and Allied Health Care, Monash University, Level 3 Building G, McMahons Road, Frankston, VIC 3199, Australia

**Keywords:** mHealth, malnutrition, upper gastrointestinal cancer, dietetic intervention, quality-adjusted life years, behaviour change

## Abstract

Background: Delay in dietetic service provision for upper gastrointestinal cancer exacerbates disease-related malnutrition and consequently increases morbidity and mortality. Dietetic services are usually referral-based and provided face-to-face in inpatient or outpatient settings, which can delay the commencement of nutrition care. The aim of this study was to provide intensive dietetic intervention close to the time of diagnosis for upper gastrointestinal cancer and assess the effect on quality-adjusted life years. Methods: A three-arm randomised controlled trial of adults newly diagnosed with upper gastrointestinal cancer was performed. A behavioural-based, individually tailored, symptom-directed nutrition intervention was provided in addition to usual care, delivered by a dietitian using a telephone (synchronously) or a mobile application (asynchronously) for 18 weeks, compared with a usual care control group. Data were collected at baseline, three, six, and twelve months post-randomisation. The primary outcome was quality-adjusted life years (EQ-5D-5L quality of life assessment tool). Data were analysed using linear mixed models. Results: One hundred and eleven participants were randomised. Quality-adjusted life years were not different in the intervention groups compared with control (telephone: mean (95% CI) 0.04 (0.43, 2.3), *p* = 0.998; App: −0.08 (−0.18, 0.02), *p* = 0.135) after adjustment for baseline, nutrition risk status, age, and gender. Survival was similar between groups over 12 months. The asynchronous mobile app group had a greater number of withdrawals compared with the telephone group. Conclusion: Early and intensive nutrition counselling, delivered at home, during anticancer treatment did not change quality-adjusted life years or survival over 12 months compared with usual care. Behavioural counselling alone was unable to achieve nutritional adequacy. Dietetic services delivered asynchronously using a mobile app had low acceptance for patients undergoing anticancer treatment. Trial Registration: 27 January 2017 Australian and New Zealand Clinical Trial Registry, ACTRN12617000152325.

## 1. Introduction

Cancers of the stomach, oesophagus, and pancreas (upper gastrointestinal, UGI) are leading causes of cancer deaths worldwide [1]. These cancers and treatments cause declines in nutritional status and quality of life (QoL) [2,3]. Weight loss prior to chemotherapy has been associated with poorer overall survival in numerous cancer types, including gastric cancer [4]. Weight loss is often a presenting feature of UGI cancers, although the time to initiation of nutrition intervention varies considerably, resulting in the inconsistent provision of dietetic services to patients [5,6,7]. In practice, referral to dietetics services occurs very late in the care pathway, enabling nutritional decline and malnutrition to become established and nutrition impact symptoms to become barriers to effective food-based interventions leading to poor QoL [3,6,7,8,9].

A systematic review of the literature found that for people with cancer (including gastrointestinal cancers), a better nutritional state is associated with better QoL [10]. There is limited high-quality evidence to determine whether nutrition intervention during cancer treatment improves QoL and nutritional status in people with UGI cancer [11,12,13,14,15]. Rigorous randomised controlled trials to determine the optimal timing, duration, and intensity of nutrition intervention during cancer treatment are needed [5]. The rapid growth in different approaches to delivering health interventions provides new service delivery opportunities that may be advantageous over more traditional approaches such as face-to-face and telephone follow-up. For example, in terms of accessibility, face-to-face services require both patient and dietitian to be available at the same time and in the same place which may be difficult during cancer treatment [7,16]. Telephone-based services may be more accessible, but they still require patients and therapists to be available at the same time. Mobile applications allow patients and clinicians to connect asynchronously and can also be structured to facilitate the application of behaviour change techniques such as goal setting and review, delivered at a time the patient wishes to access the information [17,18]. A recent review identified that asynchronous delivery modes can be used to elicit behaviour change to improve health outcomes in cancer patients compared with usual care or no intervention [19]. However, there has been no direct comparison of the same intervention delivered synchronously compared with asynchronously, and compared with usual care [19].

The objective of this trial was to deliver early and intensive dietitian-led nutrition counselling to people commencing treatment for UGI cancer, and assess the effects on quality-adjusted life years (QALY) lived compared with usual care.

## 2. Materials and Methods

### 2.1. Study Design

A three-arm randomised controlled trial was performed. Ethical approval was granted by the Monash Health Human Research Ethics Committee (14 October 2016 HREC/16/MonH/290). Site-specific authorisation was granted for all sites (Monash Health, Cabrini Health and Peninsula Health). Participants provided informed verbal consent to participate. The trial was registered prospectively on the Australian and New Zealand Clinical Trial Registry (Trial ID: ACTRN12617000152325 27 January 2017). The detailed protocol has been published [20].

### 2.2. Participants and Setting

Participants were newly diagnosed (<4 weeks) with UGI cancer and planned to commence surgical and/or medical (chemo- and/or radiotherapy) cancer treatment. Participants were recruited from health services (public and private) in southeast Victoria, Australia.

### 2.3. Eligibility

Patients who had received urgent surgical treatment prior to recruitment were eligible. Commencement of chemotherapy or radiotherapy prior to recruitment deemed the person ineligible.

### 2.4. Recruitment

Recruitment occurred between April 2017 and July 2019. Individuals were screened for eligibility by surgeons, ward dietitians, multidisciplinary team discussions, or screening of weekly outpatient upper gastrointestinal clinic list. Eligible participants were contacted either in person or via telephone and invited to participate.

### 2.5. Randomisation and Blinding

Randomisation was completed by an independent biostatistician. A permuted block randomisation with two group stratification (Malnutrition Screening Tool (MST) score of <3 or ≥3 [21]) was performed using computer-generated random numbers (STATA version 14, StataCorp LP, College Station, TX, USA). Researchers conducting recruitment, data collection, and data analysis were blinded to group allocation. Group allocation was concealed in opaque sealed envelopes and revealed to the participant by the research dietitian conducting the nutrition intervention.

### 2.6. Usual Dietetic Care and Control Group

Dietic care was referral-based, reliant on the physician identifying the need. Participants who attended a UGI outpatient service were screened for nutritional risk via MST [21], with a score of ≥3 triggering a referral to a general dietetic outpatient clinic, which required participants to wait up to several weeks to access the service [3]. Participants who had an inpatient admission, which could be up to 6 weeks after the time of diagnosis [22], were screened by nursing staff, and if nutritional risk was indicated they were referred to the dietetics service; or immediate referral if admission was for major upper gastrointestinal and hepatobiliary surgeries. The control group received usual care.

### 2.7. Intervention Groups

The intervention commenced as early as possible from the time of diagnosis and ran as a ‘centralised’ dietetics service for 18 weeks additional to usual care. The intervention was delivered by an experienced oncology dietitian as reported elsewhere [20]. Briefly, tailored nutritional recommendations were co-developed, based on medical history and nutrition impact symptoms. Goals were set and guidance was provided on how to perform the behaviour; goal achievement was monitored. Nutrition intervention was via telephone (synchronously) or an internet-enabled mobile app ‘myPace’ (random allocation). myPace was designed underpinned by behaviour change theory [23] and enables participants to self-monitor their goal attainment and body weight. For both groups, reviews were planned weekly or fortnightly (depending on need). If participants contacted the dietitian in addition to scheduled reviews or sought a private consultation with a dietitian external to the study, this was documented. Further information is reported in Supplementary File S1 according to the TIDiER checklist.

### 2.8. Community Involvement

Two community members contributed to the advisory committee over the duration of the study, from planning through to results discussion (2016–2020). Through reflecting and sharing their experience of cancer treatment, they contributed to procedures of recruitment, intervention provision, data collection procedures and interpretation of results.

### 2.9. Data Collection

Outcome data were collected by a blinded assessor at baseline, three, six, and twelve months via telephone or face-to-face.

### 2.10. Primary Outcome

QALY were calculated using the EuroQol 5D-5L instrument (EQ-5D-5L) [24] using the area under the curve calculation approach. Utility values were determined at each time point before converting to the QALY lived, using the approach reported by Norma et al. 2013 [25] (model D); a utility value of zero was given from the date of death onwards.

### 2.11. Secondary Outcomes

Cancer-specific quality of life was measured using the EORTC-C30 scale [26,27]. Date of death was recorded over the 12-month follow up period to examine survival. Nutritional status was measured using the short form version of the Patient-Generated Subjective Global Assessment (PG-SGA_SF_); this is a change from the published protocol [20] as the physical examination component of the PG-SGA was not possible due to the majority of follow up data collection not being conducted in-person [28,29,30]. Participants reported on their weight and weight history, food intake history, nutrition impact symptoms, and activities of daily living and function. Changes in self-reported body weight were also assessed and cross-referenced with available medical records for accuracy.

### 2.12. Sample Size Calculation

Pilot data informed the sample size estimate [3], with a smaller standardised effect size estimate (0.70) for the present study, which estimated that *n* = 33 participants per group were required to attain 80% power for comparisons with the control group using a two-tailed alpha of 0.05. We inflated this to *n* = 37 per group to account for potential drop-outs.

### 2.13. Data Analysis

Analysis was performed in STATA version 14 (StataCorp LP, College Station, TX, USA). Participants who died were ascribed a score of zero from their recorded date of death. Multiple imputation was used to replace other missing individual data points for conducting comparisons in mean QALY per participant between groups [31,32,33]. Supplementary File S2 contains a detailed analysis to examine the patterns of missing data. The missing data were likely to be missing not at random; therefore, sensitivity analyses were conducted following multiple imputation, examining the potential impact on the base-case multiple imputation result. These sensitivity analyses systematically varied the magnitude of the imputed utility values by +0.1, −0.1, −0.25, and −0.5 (reflecting that half of the missing utility values were likely to be missing because they were low/participants were very unwell). Groups were compared using regression analyses adjusted for baseline EQ-5D utility values, age, gender, baseline PG-SGA short form scores, and cancer location (oesophageal, gastric, and pancreatic). QALY data from individual participants were censored at the last available measurement if the participant was lost to follow-up or withdrew from the study.

Survival was assessed using Cox proportional hazards analysis, with adjustment for age, gender, baseline PG-SGA short form scores, and cancer location. Other secondary outcomes were compared between groups using linear mixed model analyses, adjusting for baseline values of the secondary outcome and age, gender, baseline PG-SGA short form scores, and cancer location.

## 3. Results

### 3.1. Participants

**Figure 1:**
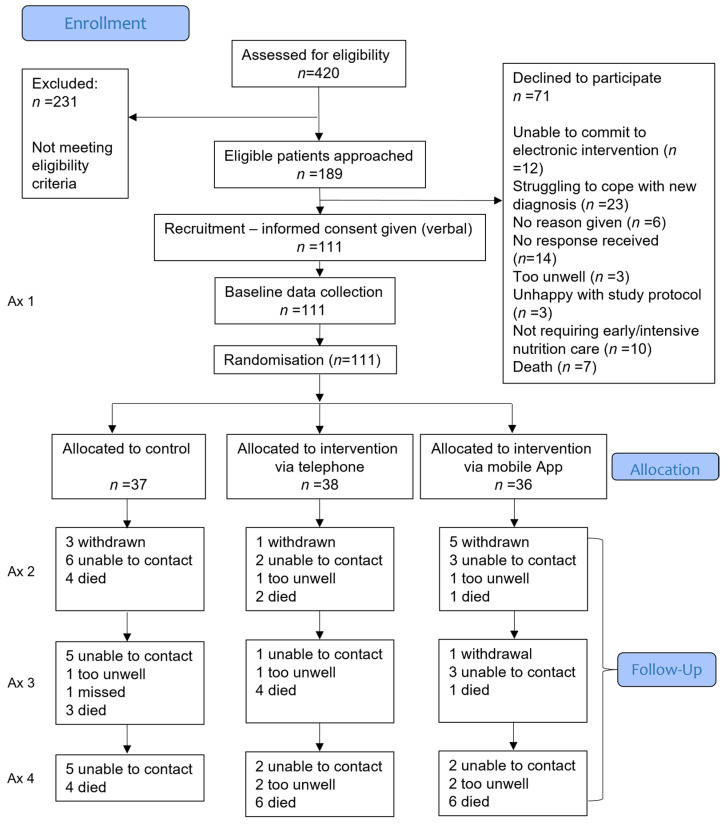
Flowchart of participants over the 12-month follow-up. Multiple imputation was used to replace missing individual data points (other than due to death) for conducting comparisons in mean QALY per participant between groups. Secondary data were analysed without imputation for missing data. Ax, assessment.

Of the 189 people identified as eligible, 111 consented to be randomised between April 2017 and July 2019 (Figure 1). Follow-up data collection was performed in July 2020. Participant characteristics are presented in Table 1.

### 3.2. Dietetic Contact

**Figure 2:**
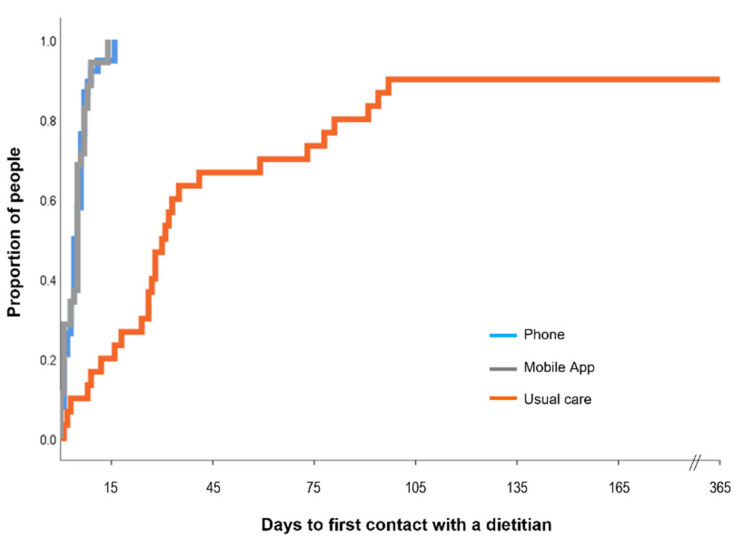
Time from randomisation to first contact with a dietitian. The first contact with a dietitian was significantly delayed in the usual care group compared with the intervention groups (telephone mean (SD) 5 (4) days, range 0–16 days *n* = 38; mobile app 5 (4) days, range 0–14 days, *n* = 33 noting that three participants withdrew prior to first contact) compared with the control group (70 (104) days, range 1–365 days, *n* = 30; *n* = 7 reported no data about contact with a dietitian). Data were censored at the end of the follow up period of 365 days.

First contact with a dietitian was markedly earlier in the intervention groups (Figure 2). The frequency of contact with a usual care dietitian at each follow-up was similar across the three groups (Table 2), demonstrating that intervention groups had earlier and more intensive nutrition intervention compared with usual care participants.

### 3.3. Numbers Analysed and Missing Data

For the primary analysis of QALY, all participants (*n* = 111) were included. For secondary outcomes, the numbers analysed are reported in the relevant tables and figures. All participants were analysed according to the group to which they were randomised.

### 3.4. Primary Outcome—Quality-Adjusted Life Years (QALY)

**Figure 3:**
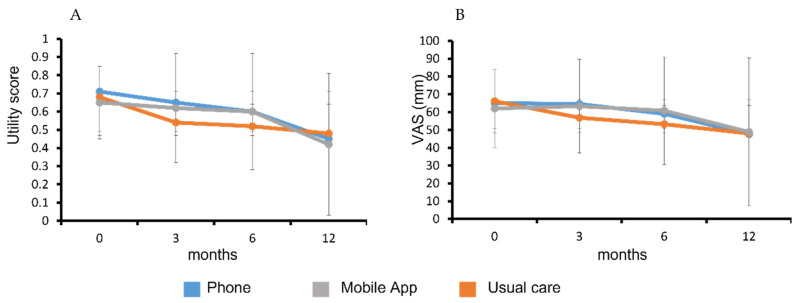
Change in health status of participants from baseline to 12-month follow-up. EQ-5D-5L utility score (**A**) and visual analogue scale of perceived health on day of assessment (**B**). Data are presented as mean (SD).

There was a declining health status in all study groups over the 12-month follow-up period (Figure 3) assessed by the EQ-5D-5L utility and VAS scores. The average QALY for each group is reported are Table 3. There were no significant differences in QALY between the intervention groups (−0.02 (−0.13, 0.08), *p* = 0.712) or compared with the control group, with adjustment for covariates (Table 4).

### 3.5. Secondary Outcomes

Assessments of quality of life using the cancer-specific EORTC QLQ-C30 were similar between groups for the global score (Table 4). Thirty-one participants died during the 12-month follow-up period (28% of all participants): eleven of the control group, twelve of the telephone group, and eight of the mobile app group. The adjusted hazard ratios were similar across the three groups (Table 4). The poorest survival was among participants with pancreatic cancer, where 18 of 44 participants died (41%); followed by oesophageal cancer, where 13 of 46 participants died (28%). None of the participants with gastric cancer died in the follow-up period. Weight loss over the 12-month follow-up was attenuated in the telephone group compared with the mobile app group (*p* = 0.031) and compared with the control, albeit not significantly (*p* = 0.075) (Table 4). Nutritional status was similar between groups (Table 4).

## 4. Discussion

This is the largest randomised-controlled trial (RCT) to date investigating the effect of a dietitian-led, individualised nutrition counselling intervention on quality-of-life outcomes in people newly diagnosed with UGI cancer. Using a three-arm design to directly compare two health service delivery modes with usual care our results showed: (1) quality-adjusted life years lived were not different between the intervention and usual care groups; (2) nutritional adequacy was not achieved with intensive remote dietetic counselling alone; (3) non-face-to-face service delivery modes enable the much earlier commencement of nutrition intervention and contact with a dietitian; however, there were disproportionately more withdrawals and missing data points from participants in the mobile app group relative to the telephone group. This finding, combined with the 12 participants who refused to participate in the study for concern of being allocated to the mobile app group, potentially indicates poorer acceptance of this mode of delivery in adults newly diagnosed with UGI cancer.

In the present study, we showed that intensive nutrition counselling commencing at diagnosis and continuing for 18 weeks had no marked impact on QoL over 12 months. Only a few published studies are available to understand the impact of nutrition counselling interventions on changes in QoL in people undergoing treatment for cancer, and the results are conflicting [15,34,35,36]. An RCT tested a three-month nutrition intervention plus three-month follow-up in cancer outpatients and found no significant difference in QoL or nutritional status between intervention (*n* = 30) and control groups (*n* = 28), despite achieving a higher protein and energy intake [36]; this study also had a high withdrawal rate. In contrast, a quasi-experimental trial of a two-month nutrition intervention for people with gastric or colon cancer (*n* = 53), commencing during an inpatient stay, found improved global QoL (EORTC QLQ-C30) and improvements in scores of physical functioning and role functioning compared with the control group (*n* = 50) [35]. Similarly, a 12-week RCT in people with gastrointestinal or head and neck cancer receiving radiotherapy found that intensive nutrition counselling (*n* = 29) mitigated weight loss and improved global QoL scores and physical function scale scores (EORTC QLQ-C30) compared with the control group (*n* = 31) [34]. These previous studies had shorter intervention periods and follow-up durations compared with the present study, and there were also differences in the cancer locations and treatment phase, which may impact on QoL scores. Weight loss despite nutrition intervention in our study suggests that nutritional adequacy was not achieved. A systematic review of nutrition interventions found that few studies have achieved nutritional adequacy to prevent weight loss in cancer therapy [15]. Nutrition impact symptoms are commonly reported in people with upper gastrointestinal cancers [37] and effective management is not achieved with dietetic care alone, because medication management is necessary and beyond the scope of dietetic practice. This highlights the importance of a multidisciplinary team approach to nutrition care during active cancer treatment. Moreover, energy requirements for people with cancer receiving anti-cancer treatment remains an understudied area [38]. Larger long-term studies are needed to determine whether attenuated weight loss during cancer treatment can be achieved and, in turn, improve QALY or survival outcomes.

A benefit of the telephone and mobile app delivery modes was that they enabled earlier commencement of nutrition intervention and access to nutrition services from patients’ own homes, reducing the traditional barriers of physical clinic space, geographical location, and transportation. The ability of carers to access evidence-based information about diet and symptom management is also important to recognise in these e-health modes. Our blanket referral approach removed reliance on the clinician identification of malnutrition risk [6,8], which is sub-optimal and delays the initiation of referral [6,8]. A cost–benefit analysis of the nutrition intervention in this study compared with usual care will inform whether a blanket referral approach is a useful process to minimise the delay in referral to a dietitian. Future studies should examine the use of e-health for the multidisciplinary care management of nutrition in UGI cancer treatment.

For successful delivery using electronic health services, digital infrastructure needs to be secure, and digital platforms should be easily operational and accepted by clinicians and patients. Seventeen percent of people who declined to participate (12/71) indicated that it was because they did not wish to be randomised to the mobile app group; throughout the study, the mobile app group had a greater number of withdrawals and missing data, particularly amongst those with poorer health status. The participant’s health status and digital literacy may be barriers to accepting or engaging with an asynchronous health service, due to lack of confidence to manage their own health [39]. Previous studies have reported that asynchronous digital platforms may be suboptimal when a person’s condition changes quickly, or important questions arise requiring decision support [40]. The prospect of learning new technology platforms at the same time as coming to terms with a diagnosis of cancer may be too overwhelming, which is supported by qualitative data from our participants who felt that their age and skill level were barriers to learning the mobile app platform after diagnosis [41]. Health service delivery needs to be patient-centred; therefore, more work needs to be undertaken to understand how digital health services should be designed to optimise the acceptance and engagement of patients, or even re-directed to enhance support to carers.

A strength of this study is that it directly compared synchronous telephone counselling with asynchronous mobile-app-delivered counselling of an individually tailored nutrition intervention in people with UGI cancer. It commenced prior to the COVID-19 pandemic, which triggered the rapid adoption of telehealth services. The high number of withdrawals from the mobile app group is a limitation, but also importantly demonstrates the preferences of people receiving health care. This study gives a better, more pragmatic representation of the likely uptake of this approach if used in a real-world setting than other designs where mobile apps are the only intervention option. The 12-month follow-up is a strength of this study compared with similar published literature.

The generalisability of standard care in the participating health services may be limited to other similar health services, and areas where the cancer care treatment pathways are less developed may have shown greater benefit from the nutrition intervention. Other limitations are that recruitment was limited to one area of Melbourne and English-speaking participants, which may not reflect the broader demographic of this patient population.

### Implications for Practice

Participants in both intervention groups were receiving nutrition care following best practice guidelines and the commencement of nutrition intervention was earlier than achieved with standard care and more frequent; however, only participants in the telephone group showed some attenuation of weight loss. Escalation to supplementary enteral or parenteral nutrition support was possible in this study through the usual nutrition care pathway during the intervention period; however, our results suggest that greater use of this type of nutrition support may be necessary to achieve nutritional adequacy. There is evidence that there are gaps in dietetic service provision in Australia which may have delayed this action [42]. Interventions for people with UGI cancer who are at very high risk of nutritional decline may require a more clinical, prescriptive approach to nutrition support, rather than intensive nutrition counselling alone, to achieve nutritional adequacy and minimise symptoms [42,43].

## 5. Conclusions

Early and intensive nutrition intervention using behavioural-based nutrition counselling delivered at home, for people newly diagnosed with UGI cancer, did not change QALY or survival during a 12-month follow-up compared with usual care. Behavioural counselling alone was unable to restore body weight to pre-diagnosis levels. The optimal management of nutrition impact symptoms requires a multidisciplinary approach to optimise the medication management of symptoms and discuss options for enteral feeding. Dietetic services delivered using e-health methods enabled the earlier commencement of nutrition intervention compared with what was achieved with usual face-to-face care. High engagement was achieved with telephone delivery; however, asynchronous delivery using a mobile app had low acceptance for patients undergoing anticancer treatment.

## Figures and Tables

**Table 1:** Participant demographics at randomisation.

	Control (*n* = 37)	Telephone (*n* = 38)	Mobile App (*n* = 36)
Age—mean (sd)	63.2 (9.9)	67.5 (10.3)	66.6 (9.7)
Gender—*n* (%)			
Male	23 (62)	25 (66)	26 (72)
Female	14 (38)	13 (34)	10 (28)
Tumour location—*n* (%)			
Oesophageal	13 (35)	16 (42)	17 (47)
Gastric	8 (22)	4 (11)	9 (25)
Pancreatic	16 (43)	18 (47)	10 (28)
Clinical stage of cancer—*n* (%)			
Resectable	16 (43)	15 (39)	18 (44)
Borderline resectable	2 (5)	1 (3)	3 (5)
Locally advanced	12 (32)	12 (32)	9 (30)
Metastatic	7 (19)	10 (26)	6 (21)
Height—mean (sd)	168.9 (10.7)	170.7 (8.9)	171.6 (9.3)
Weight—mean (sd)	75.0 (20.0)	71.9 (12.7)	76.4 (14.7)
EQ-5D-5L—median (IQR)			
Mobility	1 (1, 2)	1 (1, 1)	1 (1, 2)
Personal care	1 (1, 1)	1 (1, 1)	1 (1, 1)
Usual activities	1 (1, 3)	1 (1, 1)	1 (1, 3)
Pain or discomfort	2 (1, 3)	2 (1, 2)	2 (1, 3)
Anxiety or Depression	2 (1, 2)	1.5 (1, 3)	2 (1, 2)
EQ-5D-5L-utility score—mean (sd)	0.68 (0.19)	0.71 (0.23)	0.65 (0.20)
EQ-5D-5L visual analogue scale mean (sd)	66.16 (20.27)	65.04 (22.9)	62.08 (22.01)
First language—*n* (%)			
English	33 (89)	33 (89)	30 (86)
Familiarity with technology *n* “yes” (%)			
Do you use email?	33 (89%)	29 (76)	29 (81)
Do you have a smartphone?	30 (81%)	32 (84)	30 (83)
Do you have a tablet device?	16 (43%)	25 (66))	21 (58)
Do you feel confident to communicate with your health professional using electronic messages from your smartphone or tablet device?	33 (89%)	31 (82)	26 (72)
Do you regularly (at least once per day) use your smartphone or tablet device for purposes other than receiving or making phone calls?	30 (81%)	25 (66)	26 (72)
PG-SGA_SF_ score—mean (sd)	8.4 (6.5)	8.5 (6.2)	8.5 (6.5)
EORTC QLQ-C30 score—mean (sd)			
Global health	59.32 (25.72)	63.41 (26.17)	61.22 (24.60)
Physical functioning	79.28 (22.21)	81.23 (20.08)	77.22 (25.67)
Role functioning	63.51 (36.61)	67.54 (34.43)	65.28 (35.04)
Emotional functioning	70.49 (21.02)	72.15 (21.26)	73.38 (25.34)
Cognitive functioning	83.33 (21.52)	85.09 (18.90)	76.85 (23.66)
Social functioning	72.52 (29.71)	71.49 (30.49)	74.07 (32.96)
Fatigue	38.74 (25.48)	35.38 (29.21)	42.90 (31.44)
Nausea and vomiting	10.81 (21.59)	11.84 (20.47)	11.11 (18.26)
Pain	27.93 (27.51)	25.44 (29.70)	29.63 (33.36)
Dyspnoea	8.10 (18.27)	13.16 (23.94)	12.96 (18.30)
Insomnia	46.85 (36.39)	28.95 (29.17)	31.48 (29.76)
Appetite loss	26.13 (30.60)	35.09 (34.61)	28.70 (35.77)
Constipation	19.82 (29.89)	17.54 (28.72)	22.22 (31.87)
Diarrhoea	10.81 (23.64)	7.02 (22.13)	4.63 (19.76)
Financial difficulties	10.81 (22.30)	15.79 (29.75)	16.66 (40.19)

EQ-5D-5L, EuroQol 5D-5L instrument; EORTC QLQ-C30, European Organisation for Research and Treatment of Cancer Quality of Life Question—Core 30; PG-SGA, Patient-Generated Subjective Global Assessment; sd standard deviation.

**Table 2:** Frequency of contact with a dietitian as part of usual care *.

	Control			Telephone			Mobile App		
	3 Months (*n* = 26)	6 Months (*n* = 20)	12 Months (*n* = 18)	3 Months (*n* = 32)	6 Months (*n* = 28)	12 Months (*n* = 21)	3 Months (*n* = 26)	6 Months (*n* = 24)	12 Months (*n* = 17)
Dietitian contact prior to this follow-up—*n* “Yes” (%)	23 (88)	12 (60)	11 (61)	21 (66)	15 (54)	6 (29)	16 (61)	16 (67)	8 (47)
Median number of contacts with dietitian (range)	2.5 (0–13)	2.5 (0–26)	1 (0–15)	2 (0–14)	1.5 (0–23)	0 (0–2)	2 (0–7)	2 (0–17)	0 (0–5)

* Public or private hospital as an outpatient or inpatient, or contact with a consultant dietitian in the community.

**Table 3:** Summative outcomes by group at each follow-up.

	Control (*n* = 37)			Telephone (*n* = 38)			Mobile App (*n* = 36)		
	3 Months	6 Months	12 Months	3 Months	6 Months	12 Months	3 Months	6 Months	12 Months
QALY—mean (sd)	-	-	0.55 (0.28)	-	-	0.57 (0.28)	-	-	0.59 (0.23)
Mortality prior to this follow-up—*n* (cumulative %)	4 (11%)	3 (19%)	4 (30%)	2 (5%)	4 (16%)	6 (33%)	1 (3%)	1 (6%)	6 (22%)
*n* (% relative to baseline) ^#^	*n* = 30 (81%)	*n* = 28 (76%)	*n* = 30 (81%)	*n* = 33 (87%)	*n* = 34 (89%)	*n* = 34 (89%)	*n* = 28 (78%)	*n* = 29 (80%)	*n* = 28 (78%)
EQ-5D-5L utility score—mean (sd)	0.54 (0.37)	0.52 (0.35)	0.48 (0.42)	0.65 (0.29)	0.60 (0.35)	0.45 (0.41)	0.62 (0.30)	0.60 (0.32)	0.42 (0.39)
EQ-5D-5L visual analogue scale—mean (sd)	56.8 (29.9)	53.2 (35.6)	48.1 (41.5)	64.5 (24.1)	59.1 (32.8)	47.6 (41.4)	63.3 (26.4)	60.8 (30.2)	48.9 (41.6)
*n* (% relative to baseline) *	*n* = 26 (70%)	*n* = 20 (54%)	*n* = 18 (49%)	*n* = 32 (84%)	*n* = 28 (74%)	*n* = 21 (55%)	*n* = 26 (72%)	*n* = 25 (69%)	*n* = 17 (47%)
EQ-5D-5L—median (IQR)									
Mobility	1.5 (1, 2)	1 (1, 2)	1 (1, 1)	1 (1, 2.5)	1 (1, 2.5)	1 (1, 2)	1 (1, 2)	1 (1, 2)	2 (1, 2)
Personal care	1 (1, 1)	1 (1, 1)	1 (1, 1)	1 (1, 1)	1 (1, 1)	1 (1, 1)	1 (1, 1)	1 (1, 1)	1 (1, 2)
Usual activities	2 (1, 3)	1 (1, 2)	1 (1, 1)	1.5 (1, 3)	1.5 (1, 2)	1 (1, 2)	1.5 (1, 3)	1 (1, 2)	2 (1, 2)
Pain or discomfort	2 (1, 2)	2 (1, 2)	1.5 (1, 2)	2 (1, 3)	1 (1, 3)	2 (1, 2)	1 (1, 2)	2 (1, 2)	1 (1, 2)
Anxiety or depression	2 (1, 2)	1 (1, 2)	1 (1, 2)	1 (1, 2)	1 (1, 2)	1 (1, 2)	1 (1, 2)	1 (1, 2)	1 (1, 2)
Weight (kg)—mean (sd)	75.6 (20.3)	75.6 (17.5)	73.2 (18.4)	71.7 (11.8)	70.2 (11.7)	68.6 (13.3)	71.7 (15.6)	68.7 (14.1)	68.5 (14.1)
PG-SGA_SF_ score—mean (sd)	7.5 (5.0)	4.6 (3.6)	4.1 (4.1)	7.8 (5.7)	6.2 (5.1)	4.3 (4.7)	8.4 (6.1) ^a^	7.2 (4.0)	4.9 (3.6)
EORTC QLQ-C30 score—mean (sd)									
Global health	54.3 (25.1)	69.8 (12.2)	72.7 (15.9)	66.4 (19.7) ^b^	68.0 (28.13)	74.8 (23.8)	62.3 (24.5)	59.25 (21.10)	73.5 (20.5)
Physical functioning	70.8 (26.0)	81.3 (14.1)	86.7 (15.5)	74.0 (18.5)	75.95 (21.72)	80.6 (20.0)	73.8 (26.8)	73.33 (17.32)	82.7 (16.3)
Role functioning	48.7 (31.9)	71.7 (25.4)	78.7 (25.4)	62.0 (32.9)	68.45 (32.18)	75.4 (34.0)	59.6 (35.3)	54.67 (25.24)	77.4 (16.7)
Emotional functioning	72.8 (24.8)	80.8 (16.7)	85.6 (11.4)	82.6 (18.7)	80.65 (22.23)	84.1 (15.1)	76.3 (22.40)	73.33 (22.31)	83.8 (17.8)
Cognitive functioning	72.4 (26.6)	80.0 (17.6)	82.4 (16.6)	82.8 (19.0)	83.93 (21.98)	85.7 (20.6)	78.7 (24.8)	80 (20.41)	82.4 (21.6)
Social functioning	58.3 (41.7)	80.0 (20.7)	82.4 (27.7)	71.5 (30.2)	76.79 (25.0)	84.1 (26.1)	74.0 (32.3)	66.6 (26.8)	87.2 (21.7)
Fatigue	54.7 (26.6)	37.2 (22.0)	25.3 (22.5)	42.9 (23.5)	39.28 (23.7)	33.3 (24.3)	45.7 (26.2)	45.8 (26.3)	34.0 (26.5)
Nausea and vomiting	14.1 (16.1)	8.3 (14.8)	11.1 (21.4)	7.8 (15.8)	9.5 (12.4)	7.1 (11.3)	9.6 (14.8)	6.0 (9.5)	8.8 (19.6)
Pain	29.5 (32.1)	29.2 (24.7)	22.2 (23.6)	22.4 (30.1)	25.0 (30.6)	22.2 (22.0)	20.5 (29.9)	16.0 (21.2)	18.6 (15.5)
Dyspnoea	19.2 (30.1)	11.7 (19.6)	14.8 (23.5)	21.9 (24.8)	19.0 (24.7)	23.8 (28.2)	21.8 (23.0)	18.7 (23.7)	15.7 (23.9)
Insomnia	35.9 (35.2)	28.3 (29.2)	25.9 (21.6)	29.2 (37.6)	22.6 (27.3)	25.0 (30.3)	34.6 (40.5)	32.0 (31.1)	31.4 (34.3)
Appetite loss	38.5 (33.6)	20 (27.4)	14.8 (23.5)	32.3 (28.7)	23.8 (29.9)	20.6 (26.8)	35.9 (38.8)	26.7 (25.5)	25.5 (32.3)
Constipation	16.7 (30.2)	11.7 (24.8)	9.3 (22.3)	12.5 (22.0)	17.9 (23.1)	12.7 (19.6)	19.2 (34.2)	13.3 (25.6)	7.8 (18.7)
Diarrhoea	23.1 (32.3)	18.3 (22.9)	22.2 (30.2)	19.3 (29.5)	20.2 (27.7)	19.0 (29.0)	18.7 (27.5)	22.7 (31.5)	15.7 (24.0)
Financial difficulties	21.8 (33.9)	13.3 (29.4)	13.0 (23.3)	8.6 (19.2)	9.5 (23.8)	17.5 (34.3)	19.3 (34.0)	20.7 (33.8)	19.6 (37.4)

^#^ Sample size decreased due to withdrawal or lost to follow up. * Sample size decreased due to participant death, withdrawal, or lost to follow up. ^a^ *n* = 25; ^b^ *n* = 31; EORTC QLQ-C30, European Organisation for Research and Treatment of Cancer Quality of Life Questionnaire—Core 30; PG-SGA_SF_, Patient-Generated Subjective Global Assessment Short Form.

**Table 4:** Pairwise comparisons between groups on primary and secondary outcomes.

	Control vs. Telephone	Mobile App vs. Telephone	Mobile App vs. Control
QALY—coef (95% CI), *p*-value ^†^	0.04 (0.43, 2.3), *p* = 0.998	−0.02 (−0.13, 0.08), *p* = 0.712	−0.08 (−0.18, 0.02), *p* = 0.135
Survival—HR (95% CI), *p*-value *	0.999 (−0.45, 2.39), *p* = 0.923	0.61 (0.27, 1.74), *p* = 0.434	0.52 (0.23, 1.50), *p* = 0.265
EORTC QLQ-C30 score ^#^^,^^†^			
Global health	−4.02 (−10.4, 2.4), *p* = 0.22	−6.00 (−12.70, 0.75), *p* = 0.082	−0.67 (−7.62, 6.28), *p* = 0.850
Physical functioning	−2.75 (−9.63, 4.12), *p* = 0.433	−3.20 (−10.03, 3.63), *p* = 0.359	−2.31 (−8.29, 3.67), *p* = 0.448
Role functioning	−6.11 (−16.78, 4.56), *p* = 0.262	−6.31 (−16.16, 3.54), *p* = 0.210	−0.12 (−9.95, 9.71), *p* = 0.980
Emotional functioning	−0.88 (−8.08, 6.33), *p* = 0.812	−7.07 (−14.37, 0.22), *p* = 0.057	4.95 (−1.88, 11.78), *p* = 0.155
Cognitive functioning	−7.36 (−14.15, −0.57), *p* = 0.034	−1.60 (−8.57, 5.37), *p* = 0.652	−6.43 (−13.90, 1.04), *p* = 0.092
Social functioning	−5.38 (−16.73, 6.00), *p* = 0.353	−3.01, (−12.30, 6.28), *p* = 0.525	−4.93 (−16.54, 6.68), *p* = 0.405
Fatigue	3.08 (−5.77, 11.93), *p* = 0.496	3.28 (−5.63, 12.19), *p* = 0.471	1.47 (−7.63, 10.58), *p* = 0.751
Nausea and vomiting	0.02 (−5.68, 5.72), *p* = 0.994	−1.94 (−6.91, 3.04), *p* = 0.445	3.17 (−2.16, 8.50), *p* = 0.244
Pain	1.22 (−9.04, 11.47), *p* = 0.816	−5.87 (−15.60, 3.85), *p* = 0.237	11.63 (1.20, 22.06), *p* = 0.029
Dyspnoea	−0.76 (−9.66, 8.13), *p* = 0.867	1.26 (−7.14, 9.65), *p* = 0.769	1.67 (−6.67, 10.02), *p* = 0.694
Insomnia	−1.94 (−14.94, 11.06), *p* = 770	5.21 (−8.13, 18.56), *p* = 0.444	−2.06 (−15.12, 11.00), *p* = 0.757
Appetite loss	0.65 (−9.71, 11.00), *p* = 0.902	3.49 (−6.74, 13.72), *p* = 0.504	−2.01 (−12.71, 8.70), *p* = 0.713
Constipation	−2.44 (−12.35, 7.35), *p* = 0.625	2.75 (−7.11, 12.62), *p* = 0.584	−0.35 (−12.25, 11.56), *p* = 0.955
Diarrhoea	4.84 (−7.16, 16.83), *p* = 0.429	−0.61 (−12.53, 11.31), *p* = 0.920	3.94 (−8.47, 16.35), *p* = 0.534
Financial difficulties	6.00 (−4.96, 16.97), *p* = 0.283	8.54 (−1.37, 18.46), *p* = 0.091	−4.0 (−16.00, 8.00), *p* = 0.514
PG-SGA_SF_ score *	−0.87 (−2.69, 0.94), *p* = 0.346	0.57 (−1.42, 2.55), *p* = 0.575	−1.20 (−2.98, 0.58), *p* = 0.186
Weight ^†^	−2.43 (−5.11, 0.25), *p* = 0.075	−2.56 (−4.89, −0.23), *p* = 0.031	0.92 (−1.65, 3.50), *p* = 0.481

^#^ Multiple imputation was used to replace missing individual data points for conducting comparisons in mean QALY per participant between groups. ^†^ Adjusted for baseline value of outcome measure, age, gender, baseline PG-SGA_SF_, and cancer location. * Adjusted for age, gender, baseline PG-SGA_SF_ score, and cancer location. EORTC QLQ-C30, European Organisation for Research and Treatment of Cancer Quality of Life Questionnaire—Core 30; HR, hazard ratio; PG-SGA_SF_, Patient-Generated Subjective Global Assessment Short Form; QALY, quality-adjusted life years.

## Data Availability

The data presented in this study are available on request from the corresponding author. The data are not publicly available due to the requirement that proposed uses must have been by an ethics review board.

## Supplementary Materials

The following Supporting Information can be downloaded at: https://www.mdpi.com/article/10.3390/nu14153234/s1, File S1: TIDieR Checklist; File S2: Missing data report. References [44,45] are cited in the Supplementary File S2.
