# Supplementary material for: Effect of Early and Intensive Telephone or Electronic Nutrition Counselling Delivered to People with Upper Gastrointestinal Cancer on Quality of Life: A Three-Arm Randomised Controlled Trial
Source: Nutrients. 2022 Aug 7;14(15):3234. doi: 10.3390/nu14153234 (PMC9370208; doi:10.3390/nu14153234)
Supplement: Supplementary file 1 [file nutrients-14-03234-s001.zip › Supplementary File S1.pdf]

TIDieR Checklist

BRIEF NAME THAT DESCRIBES THE INTERVENTION

INTERVENTION GROUP 1 - Early and frequent dietitian-led nutrition counselling via telephone

WHY

The purpose of the telephone consultations was to deliver appropriate nutritional advice and strategies for managing nutrition-impact symptoms as soon as practicable following diagnosis.

WHAT

Participants randomised to the telephone intervention group requried regular access to a telephone. Nutrition advice and support was provided over the phone and tailored to each participant, informed, in part, by the treatment pathway planned by the multidisciplinary team treating the participant, and also by the needs of the participant throughout the study period. Nutrition information fact sheets were available to participants which described strategies for managing nutrition impact symptoms associated with disease progression and treatment side effects (e.g managing fatigue, nausea, vomiting). Information/resources were posted via mail or email to the participant. Participants were also be provided with forms to order oral nutrition supplements if deemed relevant.

All participants continued to receive usual nutrition care provided by their health service. For those randomised to an intervention group, participants received the intervention over 18 weeks, commencing as soon as possible after diagnosis.

WHO PROVIDED

The intervention was provided by a dietitian experienced working with the upper gastrointestinal cancer population and skilled in using behaviour change techniques.

HOW

The early and frequent nutrition support/ advice was delivered to individual participants by telephone.

WHERE

The intervention delivery occurred via telephone when the participant was at home (or at a location of their choosing) provided they had access to a telephone. Participants who had a hospital admission were not reviewed by the research dietitian at this time because dietetic input was delivered as part of 'usual care.' Dietetic reviews provided as part of the research protocol were re-commenced once the participant was discharged. The intervention timeline was not suspended during hospital admissions.

WHEN and HOW MUCH

- Initial contact within first week from date of recruitment.
- First follow-up within first week after initial contact.
- Minimum fortnightly contact (15 -30 minutes) thereafter for general check-ups or more frequent at discretion of treating dietitian over a period of 18 weeks.

TAILORING

Individual nutrition support/ advice was provided. Participants' needs vary depending on the tumour location, the treatment pathway, nutrition impact symptoms and treatment side effects experienced. Participants set weekly goals aimed at promoting nutritional intake.

INTERVENTION GROUP 2 - Early and frequent nutrition support via mobile application

WHY

The purpose of the mobile application intervention group was to deliver appropriate nutritional support and advice as soon as practicable following diagnosis.

WHAT

Participants randomised to the mobile App group received all dietetic-led nutrition intervention asynchronously via an internet-enabled mobile App 'myPace'. myPace was underpinned by behaviour change theory (26) and enabled the participants to self-monitor their pre-determined goals and goal attainment, as well as changes in weight. Automated daily reminders were set to promote behaviour change and adherence. Communication was asynchronous with planned weekly or fortnightly reviews completed by the dietitian. If participants contacted the dietitian in addition to scheduled reviews or sought a private consultation with a dietitian external to the study, this was documented. Participants could choose to use the App on a personal device such as a smartphone, or they were supplied with a tablet device (Apple iPad Air 2, California, USA) with six months wireless connectivity. If the dietitian did not receive any messages or contact from the participant and a review was missed, the dietitian made phone contact after the first missed occurrence to confirm that there were no technical issues with the mobile App or internet connectivity, but the nutrition intervention was not discussed. Nutrition advice and support was tailored to each participant that is, in part, informed by the treatment pathway planned by the multidisciplinary team treating the participant, and also by the needs of the participant throughout the study period. Nutrition information fact sheets were available to participants which described strategies for managing nutrition impact symptoms associated with disease progression and treatment side effects (e.g managing fatigue, nausea, vomiting). The mobile application enabled text message communication, and could save and store messages along with resources provided to the participant. Participants were also be provided with forms to order oral nutrition supplements if deemed relevant.

All participants continued to receive usual nutrition care provided by their health service. For those randomised to an intervention group, participants received the active intervention for 18 weeks, commencing as soon as possible after diagnosis. The participants in the mobile application group were able to access the application up to week 26, however tailored nutrition support from the dietitian was not available beyond 18 weeks.

WHO PROVIDED

The intervention was provided by the same dietitian who delivered the telephone intervention. The dietitian was experienced working with the upper gastrointestinal cancer population and skilled in using behaviour change techniques.

HOW

Early and frequent nutrition support/ advice delivered to individual participants by mobile application (myPace).

WHERE

The intervention delivery occurred via mobile App when the participant was at home (or at a location of their choosing) provided they had a device (iPad/mobile phone/computer) with internet access. The intervention timeline was not suspended during hospital admissions.

WHEN and HOW MUCH

- Initial contact within first week from date of recruitment.
- First follow-up within first week after initial contact.
- Minimum fortnightly contact (15 -30 minutes) thereafter for general check-ups or more frequent at discretion of treating dietitian over a period of 18 weeks.

TAILORING

Individual nutrition support/ advice provided. Participants' needs vary depending on the tumour location, the treatment pathway, nutrition impact symptoms and treatment side effects experienced. Participants set weekly goals aimed at promoting nutritional intake.

MODIFICATIONS

The myPace Mobile App temporarily went offline during the intervention study which required some participants (n=3) to receive dietetic intervention reviews to be completed via email.

HOW WELL

A process evaluation has occurred alongside this RCT to assess the mechanisms of action. The protocol for this evaluation is available here: Furness, K., Huggins, C.E., Hanna, L. et al. A process and mechanism of action evaluation of the effect of early and intensive nutrition care, delivered via telephone or mobile application, on quality of life in people with upper gastrointestinal cancer: a study protocol. BMC Cancer 18, 1181 (2018). https://doi.org/10.1186/s12885-018-5089-8
