# Supplementary material for: Effect of Early and Intensive Telephone or Electronic Nutrition Counselling Delivered to People with Upper Gastrointestinal Cancer on Quality of Life: A Three-Arm Randomised Controlled Trial
Source: Nutrients. 2022 Aug 7;14(15):3234. doi: 10.3390/nu14153234 (PMC9370208; doi:10.3390/nu14153234)
Supplement: Supplementary file 1 [file nutrients-14-03234-s001.zip › Supplementary File S2.pdf]

TEND study, missing data report.

Preface

Data collection for the TEND study was completed in 2020. A plan for analysis by a statistician blinded to group allocation was described in our study protocol. Investigator Prof Terry Haines has undertaken this role. When this report was written the groups were concealed and referred to as groups 1, 2 or 3. Group allocation was revealed at the group investigator outcomes meeting. This had initially been scheduled in August 2020. However, this meeting was delayed due to issues with data cleaning and the appearance of an amount of missing data that may have a direct impact on study outcomes. This is of particular relevance to the primary outcome measure. Unlike other outcomes in this study which can be adequately managed using linear mixed model analysis approaches, our primary outcome of quality-adjusted life years (QALY) lived calculated using an Area Under the Curve approach requires data to be represented at each time-point for each participant. Without this there is propensity for biased results to be estimated.

This report has been amended to refer to the groups by intervention classification: phone (group 1), usual care (group 2) and mobile App (group 3).

In the following report, we have followed the approach recommended in Faria et al. 2014. We have also drawn guidance regarding execution of multiple imputation from van Ginkel et al. 2018 and Dong et al. 2013. Further we comply with guidelines from our protocol paper Hanna et al. 2018, specifically:

"QALY data from individual participants will be censored at the last available measurement if the participant is lost to follow-up or withdraws."

and

"The primary outcome will be health-related quality of life, converted to quality-adjusted life years lived. Multiple imputation will be used to replace missing individual data points for conducting comparisons in mean QALYs per participant between groups."

Finally, it is important to note that for utility-based QALY data, a person is ascribed a score of zero from the date at which they pass away.

Data setup

The initial dataset was structured so that each participant had 4 rows of data. Each row represented data from one assessment. The intended dates of each assessment were initial assessment, 3 months, 6 months and 12 months from the date of completion of the initial assessment.

Where a participant passed away during the follow-up period, the date of death was noted. If a participant died shortly after the scheduled date of an assessment, but that assessment was not completed, then that assessment was entered as being missing. The date of death was then used as the date of the following assessment and a value of zero entered in for the utility score. This means that the Area Under the Curve approach using utility values from individual assessment points would not over or under-estimate the QALY value for that participant had this approach not been used.

Where a participant withdrew from the study during the follow-up period, the date of withdrawal was noted. All scheduled assessments following the date of withdrawal were treated as missing. Where people withdrew from the study but also died during the 12-month follow-up period, they were treated as a withdrawal with missing data thereafter even though we were aware they passed away. This impacted on 1 participant in group 1 'phone' (one assessment coded as missing) and 2 participants in group 3 'mobile App) (3 assessments coded as missing).

A subset of the overall dataset was used to undertake the investigation of missing data. The dataset used for these analyses was built using only the primary outcome (variable name: eq_5d_vas), a variable coding for number of days since the initial assessment (to help declare data as panel data – variable name: days_since_first_day), a mortality variable coding for whether the participant passed away during follow-up (variable name: died_in_f_up) and whether the participant withdrew from the study (variable name: withdrawer). These were include to examine if they are auxiliary variables correlated with missing utility data to improve the precision of imputation estimates. Variables pre-specified as covariate adjustments for the primary analysis (age gender cancer_location baseline_pgsga baseline_util), along with participant_id, group allocation and assessment number variables were also included.

Analysis preparation in Stata v.15.

Analysis commands and output are displayed in `Courier New / fixed width` font.

I first declared the dataset to be panel data.

```
. xtset participant_number days_since_first_day, daily
       panel variable:  participant_number (weakly balanced)
        time variable:  days_since_~y, 01jan1960 to 31dec1960, but with gaps
                delta:  1 day
```

I then described the relevant variables to be imputed (utility eq_5d_vas) or regular (all remaining variables).

```
. mi set mlong

. mi register imputed utility
(65 m=0 obs. now marked as incomplete)

. mi register regular assessment participant_number group died_in_f_up withdrawer age gender
cancer_locatio
> n b_weight baseline_pgsga baseline_vas baseline_util days_since_first_day > gender
cancer_location b_weight baseline_pgsga baseline_vas baseline_util. mi query
(data not mi set)
```

Missing data pattern analysis

I then check the missing variable frequency and patterns.

```
. mi misstable summarize, all showzeros
                                                           Obs<.
                                                +------------------------------
               |                                | Unique
      Variable |     Obs=.     Obs>.     Obs<.  | values        Min         Max
  -------------+--------------------------------+------------------------------
  participan~r |         0         0       444  |    111          1         162
    assessment |         0         0       444  |      4          1           4
         group |         0         0       444  |      3          1           3
  died_in_f_up |         0         0       444  |      2          0           1
    withdrawer |         0         0       444  |      2          0           1
           age |         0         0       444  |     38         38          88
        gender |         0         0       444  |      2          0           1
  cancer_loc~n |         0         0       444  |      3          1           3
      b_weight |         0         0       444  |     72         42         126
  baseline_p~a |         0         0       444  |     23          0          23
  baseline_vas |         0         0       444  |     23          5         100
  baseline_u~l |         0         0       444  |     52       .074        .941
       utility |        65         0       379  |     99      -.519        .941
  days_since~y |         0         0       444  |     78      21916       22281
  -----------------------------------------------------------------------------

. mi misstable patterns

   Missing-value patterns
     (1 means complete)

              |   Pattern
    Percent   |  1
  ------------+-------------
       85%    |  1
              |
       15     |  0
  ------------+-------------
      100%    |

  Variables are  (1) utility

. mi misstable tree

  Nested pattern of missing values
     utility
  ----------
          15%
          85
  ----------
 (percent missing listed first)
.
```

There were 38 participants who had a total of 65 missing utility assessment points. This represents 14.6% of all assessment points, and 19.5% of all follow-up assessment points. The distribution of missing utility values across different assessment points and different groups is displayed [overall frequency, n in those who died during follow-up, n in those who withdrew]:

	3-month follow-up	6-month follow-up	12-month follow-up
Group 1 (phone)	4 , 3, 1	4 , 2 , 1	5 , 0 , 1
Group 2 (usual care)	7 , 3 , 3	9 , 1 , 3	7 , 0 , 3
Group 3 (mobile App)	9 , 1 , 5	9 , 1 , 6	11 , 0 , 6

Reasons recorded for assessments being missing other than death or withdrawal were:

1) Too unwell to complete assessment.

   This applied to 8 of 38 participants (4 from group 1 (phone), 1 from Group 2 (usual care), 3 from Group 3 (mobile App)). Each recorded one missed assessment for this reason. Three of these participants (each from Group 1 phone) each died within 6 months of this scheduled assessment).

2) Administrative error.
   This applied to 1 of 38 participants (from group 2 usual care) for assessment 3 where the paperwork was lost following completion but prior to data entry.
3) Unable to be contacted / calls not returned
   This applied to the remaining participants / assessments.

Implications:
The quantity of missing data would be argued by some authors as not being inconsequential (see Dong et al.). The pattern of missing data could be described as being univariate (though we consider only the primary outcome here, the broader dataset indicates that when this outcome was not recorded, other outcomes were also not recorded – the exception being with missing due to death where the primary outcome is included but other measures were missing). Missing data were partially monotone in pattern, and were more monotone where a participant withdrew from the study.
Missing data appeared distributed into group 3 mobile App > 2 usual care > 1 phone, though distribution across follow-up points was relatively even. Twenty-nine of the 65 missing utility assessment points were in participants who eventually withdrew. Eleven of the 65 missing utility assessment points were in participants who passed away during the 12-month follow-up period. There were an additional three participants who died in this period but withdrew from the study prior to death. There were additional participants who died not long after study completion, potentially contributing to missing data at the 12-month follow-up but the above table does not represent these.

Examination of factors that were correlated with missing data variable and that predict missingness

Factors that are correlated with present values of the variable with missing values can be used to help identify the type of missingness (Missing Completely At Random, Missing At Random, Missing Not At Random), and can also be useful for imputing missing values if Missing At Random). These variables are referred to as auxiliary variables:

```
 pwcorr assessment_cat1 assessment_cat2 assessment_cat3 group_cat1 group_cat2 group_cat3
days_since_first_day died_in_f_up age gender cancer_location_cat1 cancer_location_cat2
cancer_location_cat3 baseline_pgsga baseline_vas baseline_util utility b_weight

             | assess~1 assess~2 assess~3 group_~1 group_~2 group_~3 days_s~y
-------------+---------------------------------------------------------------
     utility |   0.1713   0.0269  -0.0044   0.0349  -0.0637   0.0285  -0.1671

             | died_i~p      age   gender cancer~1 cancer~2 cancer~3 baseli~a
-------------+---------------------------------------------------------------
     utility |  -0.5081  -0.0145   0.0813  -0.2011   0.0323   0.2066  -0.3864

             | baseli~s baseli~l  utility b_weight
-------------+------------------------------------
     utility |   0.3836   0.4333   1.0000  0.1576
```

Utility scores that are present appear to have moderate correlations with whether the person died during follow-up (rho= -0.51), the baseline PGSGA score (rho=-0.39), baseline Visual Analogue Scale score (rho=0.38 and baseline utility values (rho=0.43).

Variables were examined to see if they were good predictors of whether a utility score was missing. Observed variables that are correlated with missingness provide evidence that data are not Missing Completely At Random.

```
. logistic _mi_miss i.group, cluster(participant_number)

Logistic regression                             Number of obs     =        444
                                                Wald chi2(2)      =       5.23
                                                Prob > chi2       =     0.0733
Log pseudolikelihood = -180.65979               Pseudo R2         =     0.0229

                   (Std. Err. adjusted for 111 clusters in participant_number)
------------------------------------------------------------------------------
             |               Robust
    _mi_miss | Odds Ratio   Std. Err.      z    P>|z|     [95% Conf. Interval]
-------------+----------------------------------------------------------------
       group |
          2  |   1.967385    .884346     1.51   0.132     .8152134    4.747962
          3  |   2.696321   1.186169     2.25   0.024      1.13844    6.386063
             |
       _cons |   .0935252   .0301474    -7.35   0.000     .0497219    .1759176
------------------------------------------------------------------------------
Note: _cons estimates baseline odds.
```

Note that group 3 mobile App was predictive of having missing data relative to group 1 phone; group 2 usual care was almost there also. This analysis accounted for dependence in observations within participant.

```
. logistic _mi_miss i.cancer_location, cluster(participant_number)

Logistic regression                             Number of obs     =        444
                                                Wald chi2(2)      =       0.60
                                                Prob > chi2       =     0.7399
Log pseudolikelihood = -184.19728               Pseudo R2         =     0.0037

                      (Std. Err. adjusted for 111 clusters in participant_number)
---------------------------------------------------------------------------------
                |               Robust
       _mi_miss | Odds Ratio   Std. Err.      z    P>|z|     [95% Conf. Interval]
----------------+----------------------------------------------------------------
```

```
cancer_location |
              2  |   1.032497   .4035146     0.08   0.935     .4799844     2.22101
              3  |   .6622222   .3851474    -0.71   0.479     .2118099    2.070433
                 |
           _cons |   .1812081   .0480669    -6.44   0.000     .1077431    .3047652
---------------------------------------------------------------------------------
Note: _cons estimates baseline odds.

. logistic _mi_miss died_in_f_up, cluster(participant_number)

Logistic regression                               Number of obs     =        444
                                                  Wald chi2(1)      =       3.01
                                                  Prob > chi2       =     0.0830
Log pseudolikelihood = -182.74707                 Pseudo R2         =     0.0116

                   (Std. Err. adjusted for 111 clusters in participant_number)
------------------------------------------------------------------------------
             |               Robust
    _mi_miss | Odds Ratio   Std. Err.      z    P>|z|     [95% Conf. Interval]
-------------+----------------------------------------------------------------
died_in_f_up |   .5045872   .1991022    -1.73   0.083     .2328442     1.09347
       _cons |         .2   .0423549    -7.60   0.000     .1320587    .3028955
------------------------------------------------------------------------------
Note: _cons estimates baseline odds.
```

Note that this is a near significant finding indicating that those who died in follow-up were nearly half as likely to have a missing data point for this outcome prior to death than those who did not die. Bear in mind again that after the date of death, a value of zero was ascribed for these participants at each subsequent assessment point. A frequentist approach (ignoring non-significant findings) was not taken when considering whether missingness of data was likely to be correlated with observed measures as the trial was not designed to be powered to find minimally important associations of this nature. It was my view that an odds ratio of 0.5 would reasonably be beyond what is considered to be minimally important.

```
. logistic utility_flag age if _mi_m==0

Logistic regression                               Number of obs     =        444
                                                  LR chi2(1)        =       1.75
                                                  Prob > chi2       =     0.1857
Log likelihood = -171.21167                       Pseudo R2         =     0.0051

------------------------------------------------------------------------------
utility_flag | Odds Ratio   Std. Err.      z    P>|z|     [95% Conf. Interval]
-------------+----------------------------------------------------------------
         age |   .9817405   .0136344    -1.33   0.185     .9553781     1.00883
       _cons |   .4984083   .4511453    -0.77   0.442     .0845476    2.938117
------------------------------------------------------------------------------
Note: _cons estimates baseline odds.

. logistic _mi_miss gender, cluster(participant_number)

Logistic regression                               Number of obs     =        444
                                                  Wald chi2(1)      =       2.55
                                                  Prob > chi2       =     0.1106
Log pseudolikelihood = -182.34511                 Pseudo R2         =     0.0137

                   (Std. Err. adjusted for 111 clusters in participant_number)
------------------------------------------------------------------------------
             |               Robust
    _mi_miss | Odds Ratio   Std. Err.      z    P>|z|     [95% Conf. Interval]
-------------+----------------------------------------------------------------
      gender |    1.99242   .8608291     1.60   0.111      .854314    4.646696
       _cons |   .1044776   .0394508    -5.98   0.000     .0498435    .2189967
------------------------------------------------------------------------------
Note: _cons estimates baseline odds.
```

This analysis indicated that males had 1.99 higher odds of having missing data for this outcome.

```
. logistic _mi_miss baseline_pgsga, cluster(participant_number)

Logistic regression                               Number of obs     =        444
```

```
                                                Wald chi2(1)      =       0.01
                                                Prob > chi2       =     0.9286
Log pseudolikelihood =  -184.8795               Pseudo R2         =     0.0000

                     (Std. Err. adjusted for 111 clusters in participant_number)
--------------------------------------------------------------------------------
               |               Robust
      _mi_miss | Odds Ratio   Std. Err.      z    P>|z|     [95% Conf. Interval]
---------------+----------------------------------------------------------------
baseline_pgsga |   1.002177   .0243182     0.09   0.929       .95563    1.050992
         _cons |   .1683531   .0497109    -6.03   0.000     .0943801    .3003044
--------------------------------------------------------------------------------
Note: _cons estimates baseline odds.

. logistic _mi_miss b_weight, cluster(participant_number)

Logistic regression                             Number of obs     =        444
                                                Wald chi2(1)      =       0.17
                                                Prob > chi2       =     0.6814
Log pseudolikelihood = -184.68821               Pseudo R2         =     0.0011

                   (Std. Err. adjusted for 111 clusters in participant_number)
------------------------------------------------------------------------------
             |               Robust
    _mi_miss | Odds Ratio   Std. Err.      z    P>|z|     [95% Conf. Interval]
-------------+----------------------------------------------------------------
    b_weight |   .9947139   .0128425    -0.41   0.681     .9698589    1.020206
       _cons |   .2537751   .2415521    -1.44   0.150     .0392867    1.639279
------------------------------------------------------------------------------
Note: _cons estimates baseline odds.
```

Baseline PGSGA and weight did not appear to be associated with missingness.

```
. logistic _mi_miss baseline_vas, cluster(participant_number)

Logistic regression                             Number of obs     =        444
                                                Wald chi2(1)      =       4.30
                                                Prob > chi2       =     0.0381
Log pseudolikelihood = -181.28352               Pseudo R2         =     0.0195

                   (Std. Err. adjusted for 111 clusters in participant_number)
------------------------------------------------------------------------------
             |               Robust
    _mi_miss | Odds Ratio   Std. Err.      z    P>|z|     [95% Conf. Interval]
-------------+----------------------------------------------------------------
baseline_vas |   .9835467   .0078682    -2.07   0.038     .9682456    .9990897
       _cons |   .4776346   .2450201    -1.44   0.150     .1747596    1.305421
------------------------------------------------------------------------------
Note: _cons estimates baseline odds.

. logistic _mi_miss baseline_util, cluster(participant_number)

Logistic regression                             Number of obs     =        444
                                                Wald chi2(1)      =       0.19
                                                Prob > chi2       =     0.6631
Log pseudolikelihood = -184.70591               Pseudo R2         =     0.0010

                    (Std. Err. adjusted for 111 clusters in participant_number)
-------------------------------------------------------------------------------
              |               Robust
     _mi_miss | Odds Ratio   Std. Err.      z    P>|z|     [95% Conf. Interval]
--------------+----------------------------------------------------------------
baseline_util |   .6834999   .5970082    -0.44   0.663     .1233815    3.786404
        _cons |   .2217323   .1370842    -2.44   0.015     .0660056    .7448644
-------------------------------------------------------------------------------
Note: _cons estimates baseline odds.

. gen base_vas_100=baseline_vas/100

. logistic _mi_miss baseline_vas_100, cluster(participant_number)
variable baseline_vas_100 not found
r(111);

. logistic _mi_miss base_vas_100, cluster(participant_number)

Logistic regression                             Number of obs     =        444
```

```
                                                   Wald chi2(1)      =       4.30
                                                   Prob > chi2       =     0.0381
Log pseudolikelihood = -181.28352                  Pseudo R2         =     0.0195

                   (Std. Err. adjusted for 111 clusters in participant_number)
------------------------------------------------------------------------------
             |               Robust
    _mi_miss | Odds Ratio   Std. Err.      z    P>|z|     [95% Conf. Interval]
-------------+----------------------------------------------------------------
base_vas_100 |   .1903269   .1522584    -2.07   0.038     .0396783     .912951
       _cons |   .4776347   .2450201    -1.44   0.150     .1747596    1.305421
------------------------------------------------------------------------------
Note: _cons estimates baseline odds.
```

Baseline EQ-5D Visual Analogue Scale scores were associated with missingness, but baseline utility scores were not. I created a new variable by dividing the Visual Analogue Scale scores by 100 so that the interpretation of the odds ratios between this and the utility score would be somewhat comparable as in this comparison the maximum score is 1 and death is zero. The odds ratio for the Visual Analogue Scale in this revised scoring version was 0.19 compared to 0.68 for the utility scale. Both could be argued as being above a minimum important threshold.

Summary: Baseline Visual Analogue Scale scores and whether a participant died during the follow-up were identified as having associations of moderate strength with the utility values that were collected. This makes them potentially useful auxiliary variables that were not already in the analysis model that would be useful to add to the analysis model when conducting multiple imputations.

We identified Group allocation (group 3 mobile App), being male, having poorer quality of life at baseline, and not dying during the 12-month follow-up period were all associated with increased odds of missing data for the utility outcome. This provides ample evidence to suggest that missing data for this outcome are not Missing Completely At Random. The remaining options are that the data were Missing At Random (missingness is associated with observed variables but not the missing values of the variable with missing data), or Missing Not At Random (missingness is associated with the value of the missing values of the variable with missing data). If the data were Missing At Random, then analysis procedures such as Multiple Imputation or use of linear mixed models would be sufficient to generate relatively unbiased treatment effect estimates. However, if the data were Missing Not At Random, then these estimates will still be biased, and sensitivity analyses varying the value of missing data points would be useful when examining treatment effect estimates. It is impossible to actually demonstrate data are Missing Not At Random. However, we do know that:

In this study:

- Participants in Group 3 mobile App (and possibly Group 2 usual care) were more likely than those in Group 1 phone to have missing data.
- 31 of 111 participants passed away during this study.
- 8 assessment points were missing because the participant was too unwell to complete the assessments. This indicates the value of these missing utility scores would have been low.
- 3 additional participants with missing data passed away within the 12-month follow-up, but after already withdrawing from the study. This indicates the value of these missing utility scores may have been low.
- A further 18 participants passed away within 6 months of the 12-month follow-up (at the time of completing the study so this number could be higher). These participants had 12 missing assessment points. This indicates the value of these missing utility scores may have been low.
- There is a significant association between lower baseline Visual Analogue Scale scores and an increased risk of dying during the 12-month follow-up.

The combination of:

- The number of missing utility scores (19% of follow-up assessments).
- The likelihood that nearly half of the missing utility scores are likely to be missing because they were low.
- The imbalance of missing data across groups.

Leads me to the assessment that the missing data is likely to be Missing Not At Random, and likely to introduce substantial bias in the comparison between groups for the primary outcome. It is my view that sensitivity analyses should be conducted following multiple imputation examining the potential impact on the base-case multiple imputation result of systematically varying to magnitude of the imputed utility values by +0.1, -0.1, -0.25 and -0.5.

Imputation of missing data points

I used the Markov Chain Monte Carlo (MCMC) procedure which assumes that all the variables in the imputation model have a joint multivariate normal distribution. This is probably the most common parametric approach for multiple imputation (https://stats.idre.ucla.edu/stata/seminars/mi_in_stata_pt1_new/). The specific algorithm used is called the data augmentation (DA) algorithm, which belongs to the family of MCMC procedures. The algorithm fills in missing data by drawing from a conditional distribution, in this case a multivariate normal, of the missing data given the observed data. In most cases, simulation studies have shown that assuming a MVN distribution leads to reliable estimates even when the normality assumption is violated given a sufficient sample size (Demirtas et al., 2008; KJ Lee, 2010). However, biased estimates have been observed when the sample size is relatively small and the fraction of missing information is high.

Note: I set the seed at 1234 to allow reproducibility of results. 10 imputations for each row with missing data (590 rows of data in total) were added.

```
. mi impute mvn utility = died_in_f_up withdrawer age gender  baseline_pgsga baseline_vas
baseline_util days_since_first_day group_cat1 group_cat2 assessment_cat1 assessment_cat2
assessment_cat3 cancer_location_cat1 cancer_location_cat2, add(10) rseed(1234)

Performing EM optimization:
note: 65 observations omitted from EM estimation because of all imputation variables missing
  observed log likelihood =  382.38405 at iteration 1

Performing MCMC data augmentation ...

Multivariate imputation                     Imputations =       10
Multivariate normal regression                    added =       10
Imputed: m=1 through m=10                       updated =        0

Prior: uniform                               Iterations =     1000
                                                burn-in =      100
                                                between =      100

------------------------------------------------------------------
                   |               Observations per m
                   |----------------------------------------------
          Variable |   Complete   Incomplete   Imputed |     Total
-------------------+-----------------------------------+----------
           utility |        379           65        65 |       444
------------------------------------------------------------------
(complete + incomplete = total; imputed is the minimum across m
 of the number of filled-in observations.)

. tab group if _mi_m==0

      group |      Freq.     Percent        Cum.
------------+-----------------------------------
          1 |        152       34.23       34.23
          2 |        148       33.33       67.57
          3 |        144       32.43      100.00
------------+-----------------------------------
      Total |        444      100.00

. tab group

      group |      Freq.     Percent        Cum.
------------+-----------------------------------
          1 |        282       25.78       25.78
          2 |        378       34.55       60.33
          3 |        434       39.67      100.00
------------+-----------------------------------
      Total |      1,094      100.00
```

Calculation of QALYS

Data were transferred from Stata to Excel and separated into ten separate worksheets (one for each imputed dataset) to calculate QALYs for each participant.

Estimation and sensitivity analyses

This step combines the parameter estimates from the analysis output from each imputed dataset into a single set of statistics that appropriately reflect the uncertainty associated with the imputed values. Regression coefficients were the arithmetic mean of the individual coefficients estimated for each of the imputation regression models.

Sensitivity analyses were undertaken due to the high likelihood that our data were Missing Not At Random. This is because the propensity of the assessment point to be missing was likely to be related to the missing data point. In this study, several assessments were missed shortly before a participant passed away, indicating that these were likely to be low utility scores.

In the sensitivity analyses, I investigated varying the value of the imputed values by -0.1 (sensitivity analysis 1), -0.25 (sensitivity analysis 2), -0.5 (sensitivity analysis 3) and +0.1 (sensitivity analysis 4). I then proceeded to plot the values of the base analysis and the sensitivity analyses to examine the likely impact on different assumptions that could be made about the nature of the missing data.

These results are presented below:

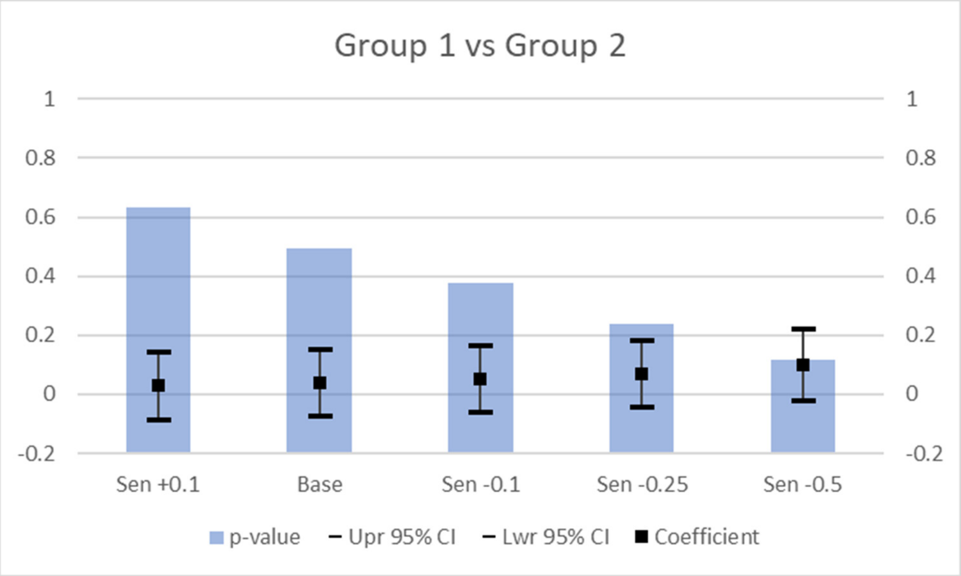

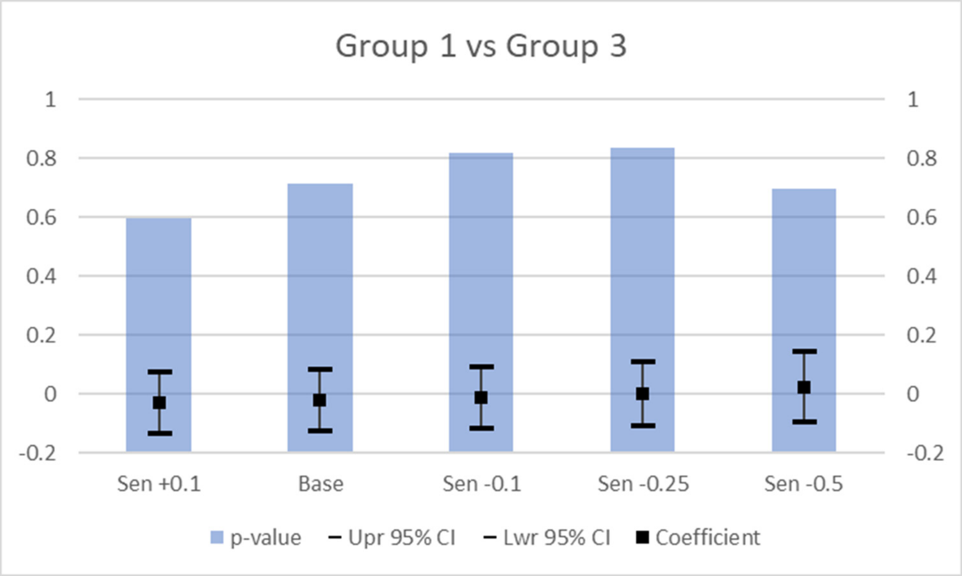

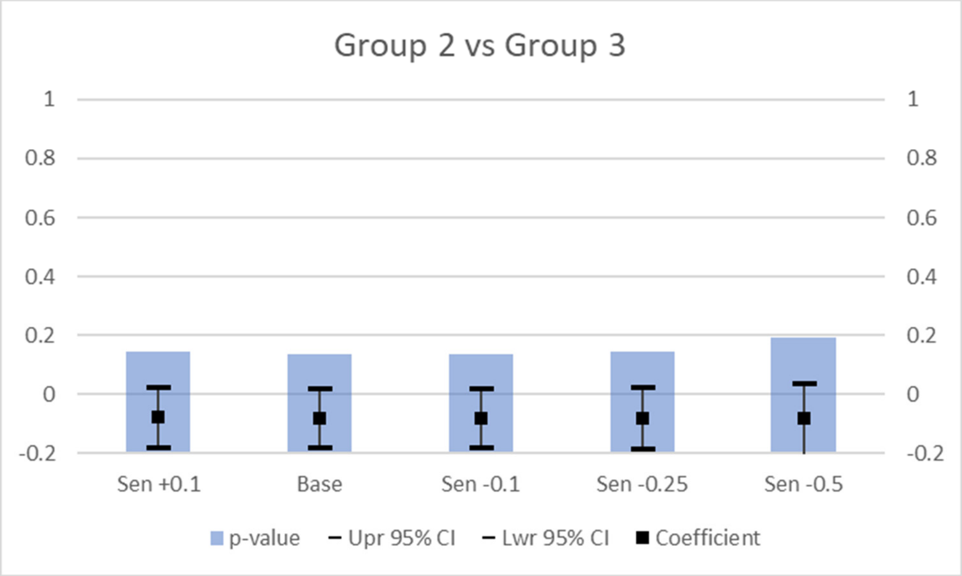

In summary: None of the base analyses identified a significant difference between groups on the primary outcome measure. Sensitivity analyses for the comparison of group 1 phone and group 2 usual care, indicated that dropping the value of imputed data by 0.5 or less did not change this result, though a clear trend was evident that dropping these imputed values by greater amount would have eventually changed the result. Sensitivity analyses comparing group 1 phone and group 3 mobile App; and group 2 usual care and group 3 mobile App did not appear to have an impact on the result.

The mean magnitudes of imputed utility values for all groups combined, divided across the different follow-up assessment points, are presented to guide whether the drop in utility examined in sensitivity analyses 2 and 3 were realistic.

Imputation set 1	3-month assessment	6-month assessment	12-month assessment
Imputed values (n, mean)	n=19, 0.53	n=20, 0.67	n=19, 0.57
Non-imputed (n, mean)	n=92, 0.60	n=91, 0.58	n=92, 0.45

As can be seen, the imputed values do not follow the anticipated trajectory value that the missing values were anticipated to follow based on what we know about the relationship between death following scheduled completion and feeling too unwell to complete the survey one as the reasons for missingness. I would therefor consider sensitivity analyses 2 and 3 to be reasonable. However, they did not change the overall study finding of no difference between groups on the primary outcome.

References

Demirtas H, Hedeker D. An imputation strategy for incomplete longitudinal ordinal data. Stat Med. 2008 Sep 10;27(20):4086-93. doi: 10.1002/sim.3239. PMID: 18338313.

Dong Y, Peng CY. Principled missing data methods for researchers. Springerplus. 2013 May 14;2(1):222. doi: 10.1186/2193-1801-2-222. PMID: 23853744; PMCID: PMC3701793.

Faria R, Gomes M, Epstein D, White IR. A guide to handling missing data in cost-effectiveness analysis conducted within randomised controlled trials. Pharmacoeconomics. 2014 Dec;32(12):1157-70. doi: 10.1007/s40273-014-0193-3. PMID: 25069632; PMCID: PMC4244574.

Hanna L, Huggins CE, Furness K, Silvers MA, Savva J, Frawley H, Croagh D, Cashin P, Low L, Bauer J, Truby H, Haines T. Effect of early and intensive nutrition care, delivered via telephone or mobile application, on quality of life in people with upper gastrointestinal cancer: study protocol of a randomised controlled trial. BMC Cancer. 2018 Jul 3;18(1):707. doi: 10.1186/s12885-018-4595-z. PMID: 29970033; PMCID: PMC6029372.

Lee KJ, Carlin JB. Multiple imputation for missing data: fully conditional specification versus multivariate normal imputation. Am J Epidemiol. 2010 Mar 1;171(5):624-32. doi: 10.1093/aje/kwp425. Epub 2010 Jan 27. PMID: 20106935.

van Ginkel JR, Linting M, Rippe RCA, van der Voort A. Rebutting Existing Misconceptions About Multiple Imputation as a Method for Handling Missing Data. J Pers Assess. 2020 May-Jun;102(3):297-308. doi: 10.1080/00223891.2018.1530680. Epub 2019 Jan 18. PMID: 30657714.
